# New Medical Journals

**Published:** 1860-04

**Authors:** 


### NEW MEDICAL JOURNALS.

The Cincinnati Medical and Surgical News, Edited by A. H. Baker, M. D.

This is what was the *Cincinnati Medical News*, now appearing in pamphlet form, with change of name, and is a very creditable publication of 32 pages, issued monthly.

The San Francisco Medical Press, Edited by E. S. Cooper, A. M. M. P.

The *Press*, the second medical journal emanating from the city of San Francisco, is a quarterly publication, containing 64 pages of printed matter, and is furnished to subscribers at two dollars per annum, payable in advance.

